# Exercise intervention regulates gut microbiota to improve type 2 diabetes: a narrative review of the mechanisms

**DOI:** 10.3389/fnut.2025.1698112

**Published:** 2025-12-24

**Authors:** Yifan Fang, Yiwen Cai, Xumin Chen, Zhiyi Lin

**Affiliations:** 1 School of Physical Education and Health Science, Guangxi Minzu University, Nanning, China; 2 College of Basic Medical Sciences, Fujian Medical University, Fuzhou, China; 3 College of Medical Imaging, Fujian Medical University, Fuzhou, China; 4 College of Physical Education and Science, Fujian Normal University, Fuzhou, China

**Keywords:** exercise intervention, gut microbiota, mechanisms, narrative review, type 2 diabetes mellitus

## Abstract

**Background:**

The gut microbiota is increasingly recognized as a key factor in the pathogenesis of type 2 diabetes mellitus (T2DM). Concurrently, exercise intervention has emerged as a promising non-pharmacological strategy for T2DM management, potentially mediated through gut microbiome modulation.

**Methods:**

This narrative review searched Web of Science, PubMed, and Embase for literature published from 1992 to the present, ultimately including 58 relevant publications. The focus was on elucidating the physiological mechanisms by which exercise modulates gut microbiota to ameliorate T2DM.

**Results:**

Our synthesis indicates that exercise training beneficially alters gut microbiota composition and function, which in turn enhances systemic insulin sensitivity and improves metabolic disturbances in T2DM. These improvements are mediated through multiple pathways, including bile acid metabolism, short-chain fatty acid production, lipopolysaccharide reduction, and branched chain amino acid catabolism. The effects of exercise on the gut microbiome are influenced by factors such as exercise intensity, duration, and type, suggesting the need for individualized regimens.

**Conclusion:**

Exercise intervention improves T2DM by modulating gut microbiota via several mechanistic pathways. Future research should prioritize personalized exercise prescriptions, larger sample sizes, integrated multi-omics approaches, and exploration of combined interventions with diet or medication to optimize T2DM prevention and treatment.

## 1 Introduction

Type 2 diabetes mellitus (T2DM) is a metabolic disorder characterized by insulin resistance and pancreatic *β*-cell dysfunction. Its pathogenesis is linked to genetic susceptibility, environmental factors, islet hypoplasia, chronic inflammation, and other determinants (1). Concurrent with global economic development and rising living standards, the worldwide incidence of T2DM has demonstrated a consistent upward trend. A recent international study on diabetes reported that the global prevalence of diabetes surged from 3.2% in 1990 to 6.1% in 2021, representing an increase of over 90% (2). Data released by the International Diabetes Federation (IDF) in 2021 indicates that the global population with type 2 diabetes has reached 537 million and is projected to rise to 643 million by 2030 (3). It has been established that exercise interventions effectively regulate blood glucose levels in patients with T2DM, thereby mitigating long-term diabetic complications (4).

In 2007, Cani et al. first demonstrated associations between gut microbiota and T2DM, revealing its role in endocrine and metabolic pathophysiology and establishing its centrality in the pathogenesis of diabetes and obesity (5). Numerous scholars have found that gut flora has the ability to alter glucose homeostasis and insulin metabolism within the body (6). Dysbiosis of gut microbiota can induce obesity and diabetes mellitus (DM) (7), and changes in the structure and function of gut microbiota can affect pathological processes such as insulin resistance, obesity, and abnormally elevated blood glucose levels (8). Moreover, the gut microbiota of diabetic patients has shown a higher relative abundance of the phylum Ascomycota and the Anaplasma phylum, and a lower relative abundance of the phylum Thick-walled bacilli, with the proportion of bifidobacteria and other recognized probiotic genera significantly lower than that of the normal population (9). Additionally, Chen et al. found through their study that Lactobacillus counts were higher and Clostridium bolbosum and Clostridium flexneri counts were lower in the feces of newly diagnosed T2DM patients compared to the healthy population (10). Lactobacillus was positively correlated with glycated hemoglobin, while *Clostridium perfringens* and Clostridium flexneri were negatively correlated with diabetic parameters. The role of gut microbiota in the pathogenesis of T2DM was systematically evaluated by Gurung et al. An inverse correlation with T2DM was observed for Bifidobacteria, Bacteroides, Clostridioides, Akkermansia, and Rotavirus, whereas Ruminococcus, Clostridium, and Rauteri demonstrated a positive correlation (11). The gut microbiota participates in the regulation of metabolic processes in T2DM through multiple pathways, including the modulation of inflammatory factors and intestinal permeability; the regulation of glucose metabolism, fatty acid oxidation, synthesis, and energy metabolism; and multi-bacterial cooperation. Furthermore, the gut microbiota is crucial for maintaining the intestinal mucosal barrier, participating in immune regulation, synthesizing essential vitamins, and fermenting undigested carbohydrates to produce short-chain fatty acids (SCFAs) (11). The occurrence and development of type 2 diabetes mellitus (T2DM) are inextricably linked to the composition of the gut microbiota, and the potential of the gut microbiota in the treatment of T2DM has been increasingly appreciated. A growing body of evidence indicates that a highly diverse gut microbiota is associated with improved health status, and regular physical exercise is positively correlated with increased microbial diversity (12). Exercise, particularly sustained aerobic or endurance training, has been demonstrated to increase the abundance of beneficial bacterial genera, such as Lactobacillus and Bifidobacterium. Lactobacillus spp. produce lactic acid, which lowers intestinal pH to inhibit the growth of pathogenic bacteria and enhances the expression of tight junction proteins (e.g., zonula occludens-1 (ZO-1)) in intestinal epithelial cells. Similarly, Bifidobacterium spp. ferment dietary fiber to produce short-chain fatty acids (SCFAs), such as acetate, propionate, and butyrate. These SCFAs contribute to gut homeostasis by upregulating tight junction proteins (e.g., ZO-1), thereby reducing intestinal permeability, and by modulating immune function through the suppression of pro-inflammatory cytokines and enhancement of anti-inflammatory responses. Furthermore, these beneficial bacteria promote the proliferation of *Akkermansia muciniphila*, a species renowned for its role in reinforcing the intestinal barrier. Collectively, these beneficial bacteria contribute to host health by maintaining intestinal pH balance, enhancing immune competence, and inhibiting the colonization of pathogenic microorganisms (13). Scholars MOTIANI et al. found that exercise training can alter systemic insulin sensitivity by regulating the gut microbiota, subsequently improving T2DM (14). Subsequently, Torquati et al. investigated the effects of different exercise intensities on the gut microbiome of patients with T2DM (15). The results indicated that moderate-intensity exercise increased the abundance of beneficial flora, while high-intensity exercise boosted the abundance of other beneficial flora. Despite the impact of exercise intensity on the gut microbiome, it did not significantly change the production of SCFAs (15). Cheng et al. utilized fecal transplantation to transfer gut flora from exercising mice to sedentary diabetic mice, and they discovered that this method optimized vascular function and metabolic status in the mice, potentially due to a connection between the gut and the vasculature (16). This presents a novel therapeutic avenue for individuals unable to engage in regular physical activity.

Considering that exercise interventions targeting the gut microbiota are emerging as a promising approach for treating T2DM, this paper reviews studies on the exercise-mediated modulation of gut microbiota to alleviate the disease. Furthermore, it provides conceptual and methodological frameworks for future research on exercise interventions aimed at Enterobacteriaceae in T2DM.

## 2 Methods

### 2.1 Search strategy

Boolean search terms included: ((exercise intervention OR physical activity) AND (gut microbiota OR gut flora) AND (type 2 diabetes OR non-insulin-dependent diabetes OR adult-onset diabetes) AND (mechanism OR physiological mechanism)) were used and searched for through Web of science, PubMed and Embase. The retrieved records were managed and screened using EndNote X9 to facilitate the removal of duplicates in the screening process.

### 2.2 Inclusion and exclusion criteria

The inclusion criteria were as follows: (1) study types: original research articles (both human and animal studies) and reviews; (2) population: individuals with or models of T2DM; (3) intervention: any form of exercise or physical activity; and (4) outcome: mechanisms linking exercise intervention, gut microbiota modulation, and improvement in T2DM.

Exclusion criteria included: (1) non peer reviewed literature (e.g., editorials, conference abstracts, theses); (2) articles not published in English; and (3) studies where the role of gut microbiota was not investigated.

Two researchers independently screened titles, abstracts, and full texts against these criteria. Any disagreements were resolved through discussion until a consensus was reached. Initially, 3,843 records were identified. After removing 1,235 duplicates, 2,608 records were screened. Following a detailed assessment, 58 articles met the inclusion criteria and were included in this narrative review.

### 2.3 Methodological rigor

To ensure the methodological quality and reporting clarity of this narrative review, its conception and writing were guided by the Scale for the Assessment of Narrative Review Articles (SANRA). The key measures taken in accordance with SANRA criteria are summarized below, with a detailed checklist provided as Supplementary material.

### 2.4 Data extraction

The following data were extracted from the 58 included studies into a standardized form: (1) authors and publication year; (2) study design (e.g., randomized controlled trial, animal study); (3) participant characteristics (e.g., human patients or animal model); (4) details of the exercise intervention (type, intensity, duration, frequency); (5) key findings related to gut microbiota changes (e.g., alterations in specific bacterial taxa); and (6) proposed or evidenced mechanisms linking exercise, gut microbiota, and T2DM improvement.

## 3 T2DM mechanisms modulated and improved by exercise intervention for gut flora

The gut microbiota, a complex community of microorganisms residing in the human gut, facilitates the breakdown of food residues through enzymatic activity, thereby supplying essential nutrients, including vitamins and amino acids, and metabolizing hazardous compounds such as nitrosamines. Based on functional roles, the gut microbiota is categorized into commensal, beneficial, and pathogenic bacteria. Beneficial bacteria enhance nutrient digestion and absorption, regulate lipid metabolism, and mitigate inflammatory responses; in contrast, pathogenic bacteria promote inflammation, compromise intestinal barrier integrity, and alter metabolic pathways, thereby detrimentally impacting host health. Dysbiosis of gut microbiota can cause a variety of gastrointestinal diseases, induce obesity, DM (7), and is closely related to thyroid disease (17), Alzheimer’s disease, autism, depression (18). Therefore, gut microbiota is an essential part of maintaining the health of the organism.

**Figure 1:**
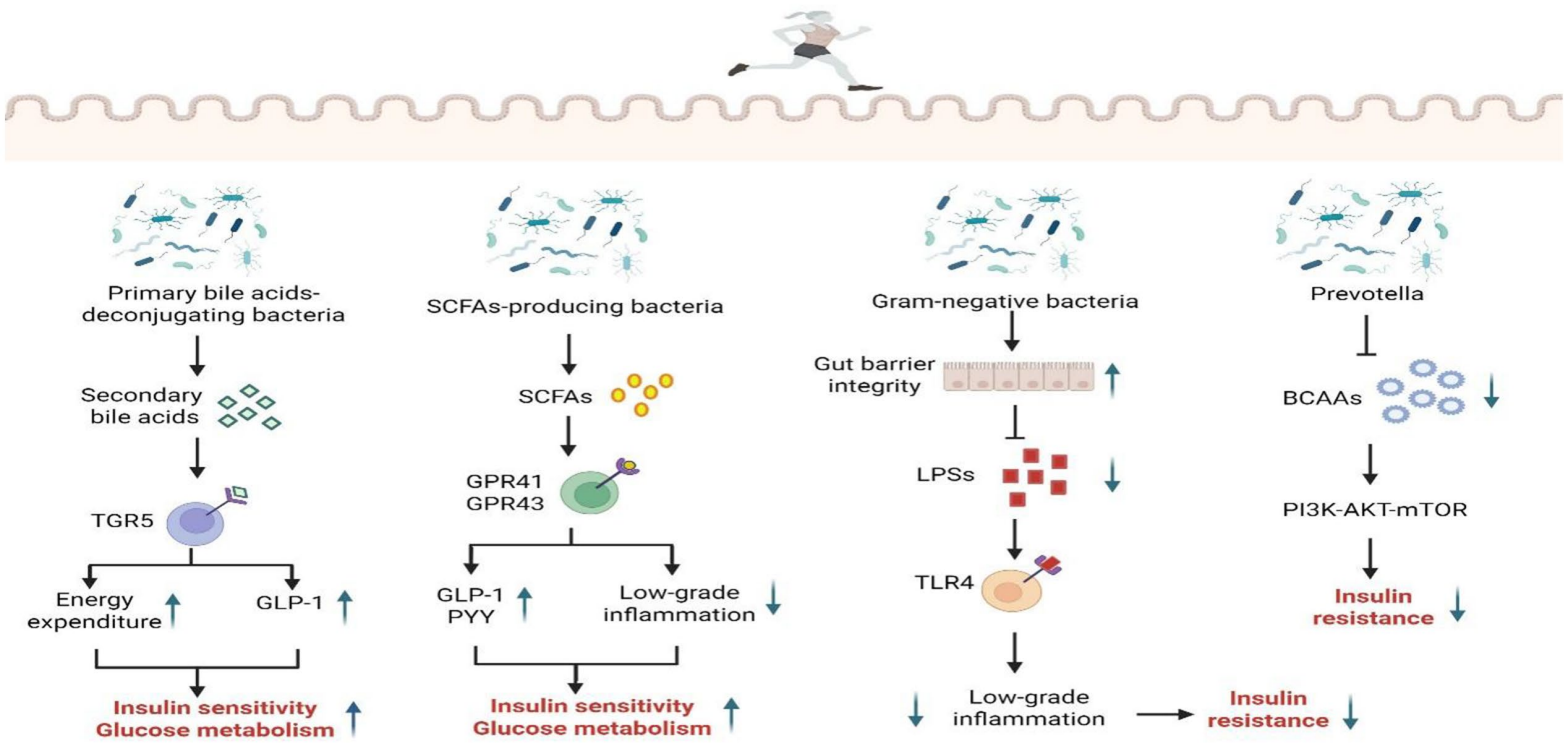
The mechanism of exercise regulating gut microbiota to improve T2DM. SCFAs, short chain fatty acids; TGR5, G protein-coupled bile acid receptor 1; GPR41, G-protein-coupled receptor41; GPR43, G-protein-coupled receptor43; GLP-1, glucagon-like peptide 1; PYY, peptide YY; LPS, lipopolysaccharides; TLR4, toll-like receptor4; BCAA, branched-chain amino acids. Created with BioRender.com.

Exercise-regulated gut microbiota intervention for T2DM offers the benefits of improving microbiota composition, stabilizing the gut microecological environment, and enhancing metabolic function. This approach represents a promising non-pharmacological strategy that circumvents the potential side effects of pharmacotherapy and mitigates risks associated with long-term drug use. Furthermore, exercise, as a sustainable lifestyle intervention, can be maintained long-term, exerting enduring effects on both the prevention and treatment of T2DM. Exercise training modulates the gut microbiota, thereby influencing systemic insulin sensitivity through multiple metabolic pathways, including bile acid metabolism, short-chain fatty acid metabolism, lipopolysaccharide metabolism, and branched-chain amino acid metabolism. This modulation constitutes a key mechanism for intervening in T2DM (Figure 1).

### 3.1 Exercise improves glucose metabolism in T2DM by regulating gut microbiota through the bile acid metabolic pathway

Bile acids (BAs) are organic acids synthesized from cholesterol in the liver, they are secreted into the intestine with bile and play a key role in fat metabolism. BAs are the main active components of bile, and their molecular structure consists of an acidic portion of a carboxyl group (COOH) and a hydrophobic portion of a steroid nucleus (also known as a steroid nucleus). This structure makes bile acids both hydrophilic and hydrophobic, thus emulsifying fat and promoting fat absorption, which can regulate the body’s glycolipid metabolic balance and energy metabolic balance. Meanwhile, abnormalities in BAs metabolism can affect insulin sensitivity, and disorders in BAs signaling pathways can lead to reduced insulin sensitivity, thereby exacerbating the development of T2DM. BAs are also involved in the regulation of glucose metabolism. By activating different signaling pathways, BAs affect lipid and glucose metabolic processes, thus regulating the abnormal glucose metabolism in T2DM.

BAs metabolism is mediated by gut microbiota. These microorganisms facilitate chemical modifications of BAs via enzymatic reactions, including binding, translocation, and 7α-dehydroxylation (19). BAs are important signaling molecules in the enterohepatic axis, which are modified by the intestinal microbiota with antimicrobial properties and can influence the growth of the gut microbiota (20). BAs play an important role in the process of fat digestion, which promotes the breakdown and absorption of fat. In diabetic patients, BAs act as regulators and activate a series of signaling pathways, thereby enhancing glucose tolerance and accelerating metabolic processes, which can help to improve the metabolism of diabetic patients. Zheng XJ et al. found that a variety of bacteria were significantly associated with BAs through animal experiments (21). Vireze et al. demonstrated that, when the structure of the gut microbiota of the subjects changed, the relative abundance of Firmicutes decreased, while the relative abundance of Bacteroidetes increased (22). The relative proportion of the thick-walled phylum decreased and the relative proportion of the anamorphic phylum increased, and this change was significantly associated with a decrease in the concentration of secondary bile acids in the feces, and there was a significant correlation between this change and an attenuation of insulin sensitivity in the subjects. Gut microbiota can convert primary BAs to secondary BAs and alter the size and composition of the BAs pool, providing complex regulation of hepatic biosynthesis and metabolism. The association between exercise, gut microbiota, and BA metabolism is supported by more than just correlative evidence. The most compelling causal evidence comes from fecal microbiota transplantation (FMT) studies. For instance, Cheng et al. demonstrated that transferring gut microbiota from exercised mice to sedentary diabetic mice was sufficient to recapitulate the metabolic benefits of exercise in the recipients, including improved vascular function and a restored bile acid profile (16). This finding strongly suggests that exercise-induced alterations in the gut microbiota are a necessary mediator for the subsequent improvements in BA metabolism and glucose homeostasis. Therefore, exercise can promote BAs secretion and increase fecal BAs levels by influencing the gut microbiota, which contributes to the optimization of gut microbiota metabolism and reorganization of the gut microbiota. Ortega-Santos et al. revealed in an animal model study that, after 12 weeks of moderate-intensity treadmill exercise training, the composition of the bile acid-associated flora of the intestinal tract of mice was positively altered (23). Primary bile acids regulate bile acid, lipid, and glucose metabolism by activating the nuclear farnesoid X receptor (FXR), while secondary bile acids activate the G protein-coupled receptor 5 (TGR5), thereby triggering the secretion of GLP-1 by enteroendocrine L cells and increasing energy expenditure in muscle (24). The incretin hormone GLP-1 stimulates insulin secretion while suppressing that of glucagon, in addition to inhibiting gastrointestinal motility and secretion. Through these dual mechanisms, appetite suppression and reduced food intake, insulin sensitivity and glucose tolerance are enhanced (25).

**Figure 2:**
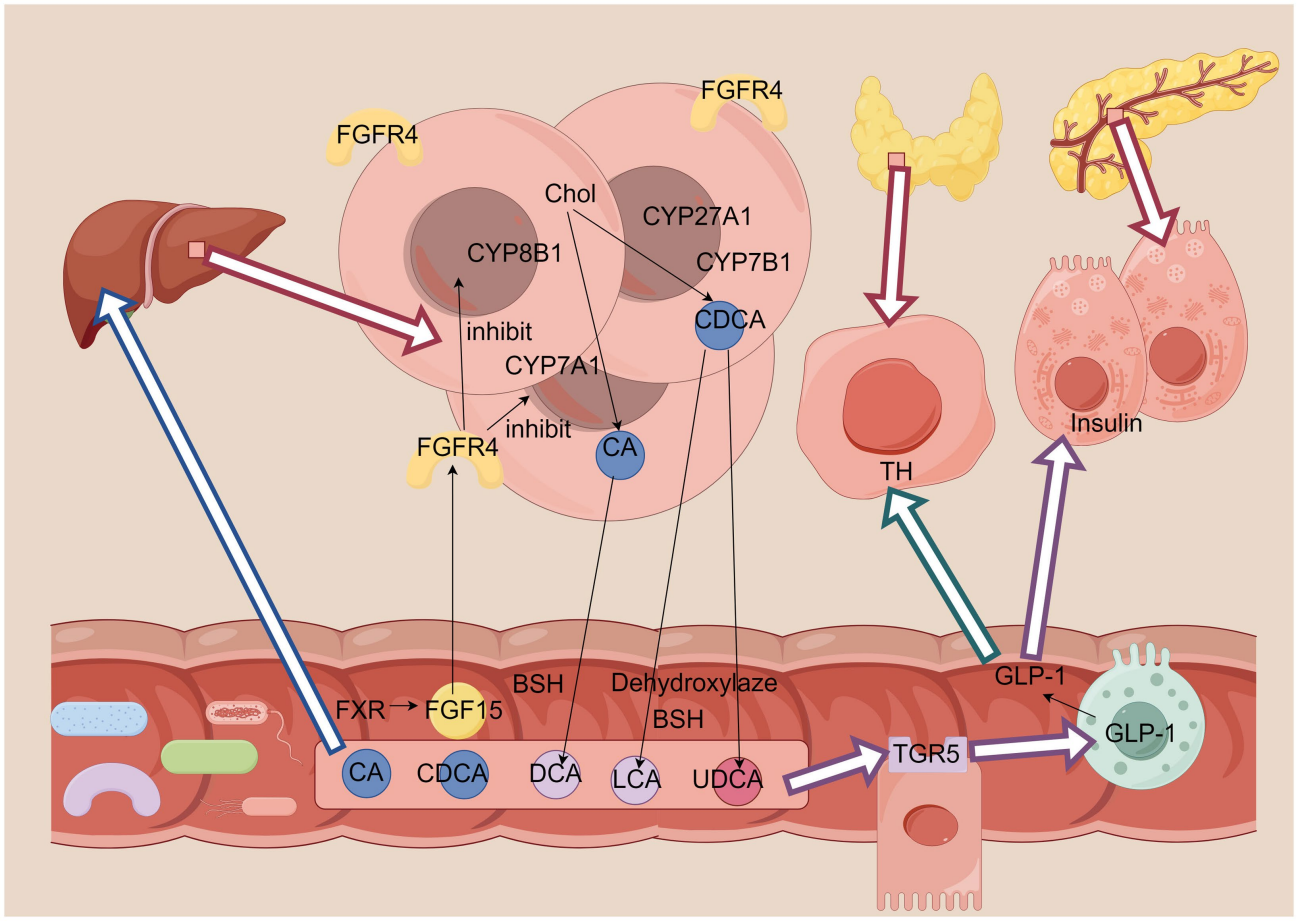
The improvement of T2DM bile acids. FGFR4, fibroblast growth factor receptor 4; CYP8B1, sterol 12-alpha-hydroxylase; CYP7A1, cholesterol 7-alpha-hydroxylase; CYP27A1, sterol 27-hydroxylase; CYP7B1, oxysterol 7α-hydroxylase; CA, cholic acid; CDCA, chenodeoxycholic acid; DCA, deoxycholic acid; LCA, lithocholic acid; UDCA, ursodeoxycholic acid; GLP-1, glucagon-like peptide I; TH, thyroid hormones. Figure drawn by Figdraw.com.

It was demonstrated by Zhang et al. that voluntary wheel running significantly improved glucose metabolism and mitigated the adverse effects of high-fat diet-induced weight gain and glucose intolerance in mice (26). Furthermore, this exercise regimen was found to induce alterations in gut microbiota composition and fecal metabolites, thereby playing a regulatory role in bile acid metabolism and secondary bile acid biosynthesis in high-fat-diet-fed mice. Exercise increases the relative abundance of beneficial microbiota in the gut, such as *Bacillus thuringiensis*, and decreases the relative abundance of Aspergillus phylum increasing the level of bile acids in the gut. At the same time, exercise leads to changes in the expression of the intestinal BAs nuclear receptor gene FXR, which helps to maintain homeostasis of cholesterol and BAs in the gut. In the liver, cholesterol is synthesized into cholic acid (CA) by the enzymes CYP7A1 and CYP8B1, which generates goose deoxycholic acid (CDCA) by the enzymes CYP27A1 and CYP7B1 (23, 27). In the intestines, intestinal bacterial bile salt hydrolase (BSH) uncouples bound bile acids, and bacterial 7α- and 7β-dehydroxylases convert the primary bile acids CA and CDCA to the secondary bile acids deoxycholic acid (DCA) and lithocholic acid (LCA), respectively. LCA. Bacteria also isomerise the 7α-hydroxyl group in CDCA to the 7β-hydroxyl group in ursodeoxycholic acid (UDCA), which is soluble and non-toxic (Figure 2). Exercise improves the gut microbiota, which can convert primary BAs into secondary BAs, primary BAs activate FXR to reduce CA synthesis and promote bile acid excretion, and secondary BAs activate TGR5 to regulate body metabolism (28), maintain glucose metabolism homeostasis, and thus improve T2DM.

### 3.2 Exercise modulates gut microbiota to improve the inflammatory state of T2DM through the short chain fatty acid metabolic pathway

**Figure 3:**
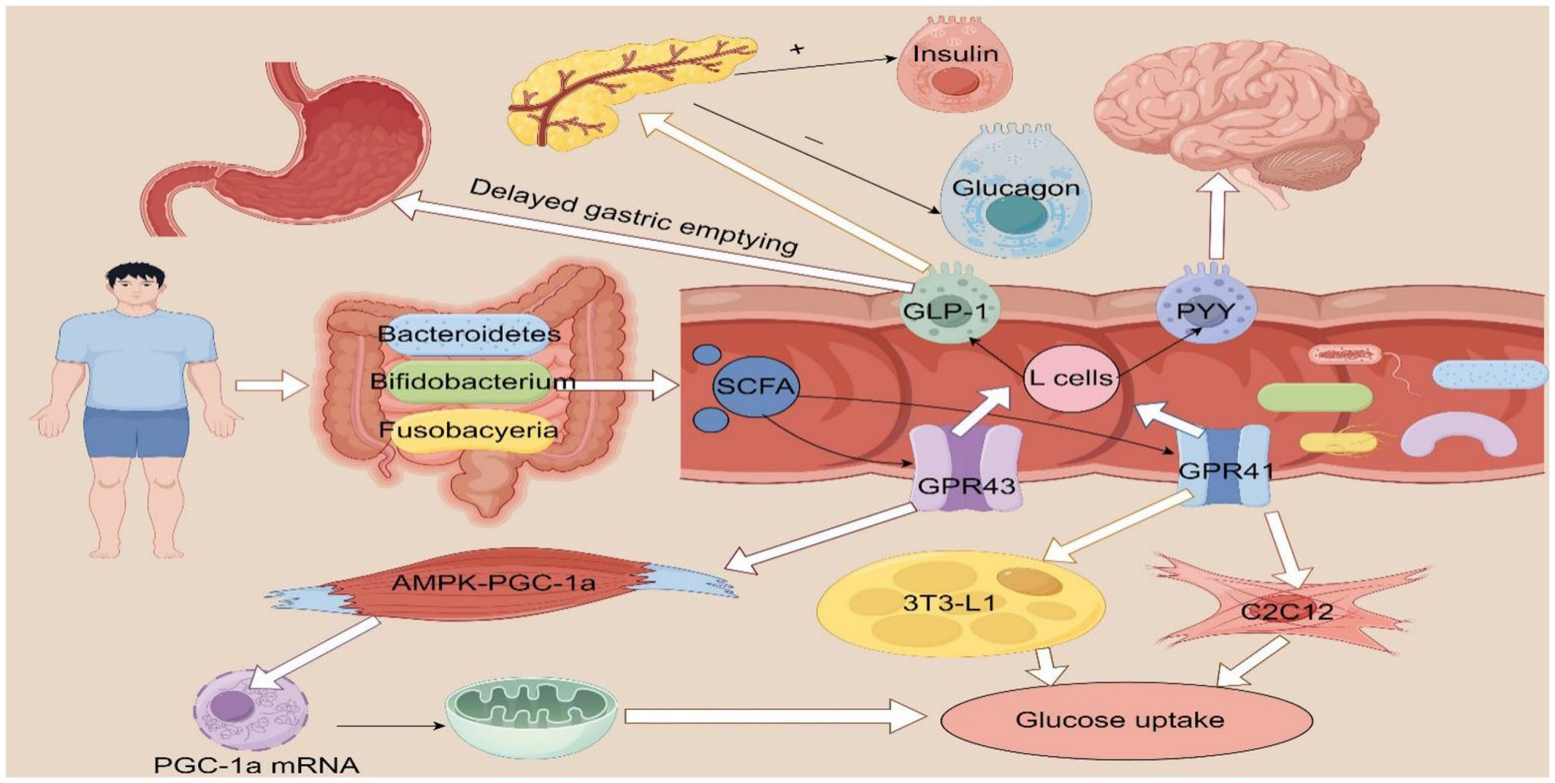
The improvement of T2DM by short chain fatty acids. SCFA, short chain fatty acids; GLP-1, glucagon-like peptide I; GPR41, G-protein-coupled receptor41; GPR43, G-protein-coupled receptor43; PYY, peptide YY; AMPK, AMP-activated protein kinase; PGC-1α, peroxisome proliferator-activated receptor gamma coactivator 1-alpha; mRNA, messenger RNA; 3T3-L1 adipocytes, 3-day transfer, inoculum 3 × 10^5^ cells; C2C12, C2C12 myoblast cell line. Figure drawn by Figdraw.com.

Exercise has been shown to increase the relative abundance of intestinal Anaerosporobacter, Bifidobacteria, and Clostridium spp., thereby enhancing SCFAs concentrations (27). This suggests that exercise-induced improvements in T2DM may be mediated through microbiota-derived metabolic products. SCFAs are the main products of gut microbiota through the decomposition of non-digestible carbohydrates, such as dietary fiber, and refer to organic fatty acids with the number of carbon atoms in the carbon chain ranging from 1 to 6, including acetate, propionate, and butyrate. Acetate is a precursor of cholesterol and fatty acids, propionate is a substrate for gluconeogenesis, and butyrate is a major energy source for colonic epithelial cells. Furthermore, intestinal gluconeogenesis is activated by butyrate, resulting in enhancements in metabolic function, including improved energy homeostasis and reduced hepatic glucose production (29). The absence of SCFA production in germ-free mice indicates that gut microbiota are essential for SCFAs biosynthesis (30). SCFAs enhance intestinal absorption, attenuate local inflammation, promote enterocyte regeneration, repair damaged mucosal barriers, and prevent ulceration and enterocolitis. Additionally, they reduce toxin translocation into systemic circulation, thereby mitigating chronic inflammation, while hepatic involvement in glucolipid metabolism lowers cholesterol synthesis (31). SCFAs stimulate L-cells in the colon to produce and secrete by recognizing and activating G-protein-coupled receptor 41 (GPR41) and G-protein-coupled receptor 43 (GPR43) in human cell lines, and the L-cells further secrete GLP-1 and peptide YY (PYY) (32). GLP-1 inhibits gastric emptying and induces satiety in the host, thus controlling the diet, and it can maintain glucose homeostasis by enhancing the secretion of insulin and decreasing the secretion of glucagon (33, 36), and it can also reduce insulin resistance, increase glucose tolerance and thus regulate glucose metabolism. PYY is produced in the human colon and regulates digestive function by reaching the brain barrier directly and activating transmission from the vagus nerve to the brainstem, resulting in a satiety effect that in turn affects appetite. Previous research has demonstrated that short-chain fatty acids (SCFAs) enhance glucose uptake in both 3T3-L1 adipocytes and C2C12 muscle cells through the activation of the GPR41 receptor (34). Furthermore, SCFAs activate the AMP-activated protein kinase (AMPK)-peroxisome proliferator-activated receptor gamma coactivator 1α (PGC-1α) pathway in skeletal muscle, leading to the upregulation of PGC-1α mRNA and protein expression. This process subsequently promotes mitochondrial biogenesis and enhances the capacity for glucose uptake and fatty acid oxidation, thereby improving blood glucose homeostasis and insulin sensitivity (35). Concurrently, bile acid synthesis is promoted by the gut microbiota. These bile acids act through the FXR and the Takeda G protein-coupled receptor 5 (TGR5) to stimulate the production of fibroblast growth factor 15/19 (FGF15/19) and the secretion of glucagon-like peptide-1 (GLP-1), respectively, which in turn enhances insulin sensitivity and glucose metabolism regulation. More specifically, FXR activation enhances fatty acid metabolism via the upregulation of PGC-1α expression, while TGR5 activation stimulates the cyclic AMP (cAMP) signaling pathway to promote fat oxidation; these mechanisms collectively contribute to improved glucose homeostasis (36) (Figure 3).

Exercise interventions can increase the abundance of butyrate-producing flora such as Clostridiaceae and Lactobacillaceae (37). Butyrate can inhibit macrophage activation and inflammatory factor production by improving the expression of altered tight junction proteins (5). McNabney et al. found that elevating the dietary butyrate content or promoting the colonization of the gut with butyrate-producing bacteria had a significant preventive and mitigating effect on IR (38). Further, a study by Vijay et al. revealed a novel link between gut microbiota composition and immune regulation, indicating that the abundance of beneficial bacteria such as Bifidobacterium and Lactobacillus was negatively correlated with the levels of the pro-inflammatory factor interleukin-6 (39). In addition, they found that exercise intervention not only increased butyrate accumulation *in vivo*, but also promoted the production of endogenous cannabinoids (e.g., arachidonamide, n-palmitoylethanolamine), and that the increase in these cannabinoids was closely associated with a decrease in tumor necrosis factor-alpha and interleukin-6, which together acted to regulate the inflammatory response in the body. This suggests that butyric acid may potentially contribute to the amelioration of T2DM by promoting the production of endogenous cannabinoids and participating in the regulatory processes of the immune system, thereby attenuating the inflammatory response.

### 3.3 Exercise regulates gut microbiota through lipopolysaccharide metabolic pathways to repair the intestinal mucosal barrier in T2DM

T2DM is a metabolic disease characterized by the presence of low-grade chronic inflammation in the body over a long period of time. Research has shown that in both T2DM patients and animal models, increased intestinal mucosal permeability and a reduction in epithelial tight junctions exacerbate inflammatory responses (40). The intestinal mucosa, a critical component of the digestive system, is primarily composed of a single layer of epithelial cells overlain by a mucus layer. Its principal function is to protect the underlying tissue from luminal pathogens and toxins. The hallmarks of intestinal mucosal injury are heightened permeability and a diminished expression of tight junction (TJ) proteins, which can lead to the translocation of bacterial LPS (41). LPS, as a key component of the outer layer of the cell wall of Gram-negative bacteria, consists of a complex of lipids and polysaccharides and is essential for maintaining the structural integrity of the bacteria. It is usually firmly attached to the cell wall, and it is only when the bacteria die that LPS has the potential to detach from the cell wall through lysis, cell disruption, and toxic effects on the host cell. Lower levels of LPS do not cause serious infections, but as an endogenous pyrogen, in humans, LPS binds to lipopolysaccharide receptor complexes on cell membranes and promotes the secretion of cytokines by inflammatory cells, which can lead to a persistent state of low-grade inflammation in the organism, triggering metabolic endotoxaemia and leading to pancreatic islet cell damage. At the molecular level, lipopolysaccharide (LPS) is a key trigger of metabolic endotoxemia, activating immune cells via the LPS-binding protein (LBP)/CD14/Toll-like receptor 4 (TLR4) signaling cascade. The ensuing inflammatory signals can disseminate to tissues including the liver, adipose tissue, and skeletal muscle, thereby promoting a state of systemic inflammation (42). Upon binding to its coreceptors, LPS activates TLR4, which initiates the nuclear factor-κB (NF-κB) and c-Jun N-terminal kinase (JNK) signaling pathways. This activation results in the excessive production of reactive oxygen species (ROS), which contributes to pancreatic *β*-cell damage and induces insulin resistance in peripheral tissues such as muscle, liver, and adipose tissue, as well as the hypothalamus (43). Furthermore, LPS disrupts tight junction (TJ) integrity by interfering with phosphorylation dynamics, leading to reduced expression of ZO-1 and consequently exacerbating inflammatory responses and intestinal mucosal damage (41). In T2DM patients, pathogenic bacterial overgrowth is frequently associated with elevated serum levels of D-lactic acid and intestinal fatty acid-binding protein (I-FABP), biomarkers indicative of compromised intestinal barrier function (44). This breach triggers a low-grade chronic inflammation that impairs glucose metabolism, potentially establishing a vicious cycle that further disrupts the gut ecosystem. Thus, LPS acts as a pivotal mediator linking intestinal barrier damage to systemic inflammation and associated metabolic disorders (45). Research indicates that long-term exercise training modulates gut microbiota composition, increasing the relative abundance of beneficial species such as *Bacteroides thetaiotaomicron*, *Bifidobacterium bifidum*, and Prevotella, while reducing systemic LPS and TLR4 levels. Concurrently, exercise enhances tight junction gene expression and suppresses pro-inflammatory cytokine release, thereby contributing to the maintenance of intestinal epithelial barrier integrity (45). One clinical study found that a six-month combined exercise program (endurance, resistance, and flexibility) for T2DM patients reduced gut pathogen colonization and decreased fecal concentrations of ZO-1 and LPS (13). Supporting these findings, fecal microbiota transplantation from wheel-running mice into germ-free recipients over 6 weeks was demonstrated to increase colonic mucus layer thickness and reduce immune cell infiltration (46). Gut flora has an important role in the body’s antioxidant system. Therefore, exercise intervention in the gut microbiota to reduce the LPS level is conducive to improving the inflammatory state, avoiding metabolic endotoxemia, reducing pancreatic islet cell damage, inhibiting the release of inflammatory cytokines, repairing the intestinal epithelial barrier, and improving the intestinal mucosal barrier in T2DM.

### 3.4 Exercise improves insulin sensitivity in T2DM by regulating gut microbiota through the branched-chain amino acid pathway

Branched-chain amino acids (BCAAs), specifically leucine, isoleucine, and valine, are amino acids that are unique because of the branched-chain structure attached to their *α* carbon atoms. Since the human body is unable to synthesize these amino acids on its own, they must be ingested through dietary protein. BCAAs play an important physiological role in triggering the secretory response of insulin and glucagon. Further, when glucagon-like peptide-1 and glucose-dependent insulinotropic polypeptide (GIP) are introduced externally via the oral route, this combination is able toprolong glucose-stimulated insulin secretion and suppress glucagon secretion, resulting in a more sustained effect on the metabolic regulation of the organism (47). In contrast, insulin reduces the turnover of BCAAs to a greater extent (48) and increases amino acid uptake relative to other amino acids (49). Shi et al. found that blood insulin sensitivity was influenced by the level of BCAAs in the body (50). Alterations in key enzyme activity during BCAA metabolism, such as reduced levels or impaired function of branched-chain aminotransferases and branched-chain ketoacid dehydrogenases, coupled with the abnormal accumulation of metabolic byproducts (e.g., acylcarnitine), may contribute to impaired insulin sensitivity in patients with T2DM. Genetic studies have revealed that variants in genes related to BCAA metabolism, particularly those encoding mitochondrial phosphatase activators like Protein Phosphatase, Mg^2+^/Mn^2+^ Dependent 1 K (PPM1K), are significantly associated with T2DM pathogenesis. The functional products of these genes within the branched-chain ketoacid dehydrogenase (BCKDH) complex are implicated in this process (51). Supporting this, an animal study demonstrated that while reduced dietary protein decreased insulin secretion in mice, supplementation with BCAAs (leucine, isoleucine, valine) restored it to normal levels (52). In humans with T2DM, genetic variants encoding BCAA synthases and transporters exhibit clustering (53). The mechanism through which elevated BCAA levels induce insulin resistance is known to involve the PI3K-AKT–mTOR signaling pathway (54). Consequently, multiple lines of evidence indicate that excessively high BCAA concentrations can promote insulin resistance. Multiple studies have established that elevated levels of branched-chain amino acids (BCAAs) can induce insulin resistance. Elevated BCAAs, particularly leucine, have been identified as upstream nutritional signals that lead to the persistent activation of the mTOR pathway. This sustained activation results in the suppression of the transcription factor Kruppel-like factor 15 (KLF15), a key regulator of glucose, lipid, bile acid, and amino acid metabolism. The downregulation of KLF15 is significant because this transcription factor is known to enhance insulin sensitivity and promote glycogen synthesis (55). Evidence suggests that exercise can induce a reduction in BCAAs through a combination of reducing the replication rate of Prevotella and promoting genes involved in the degradation of branched-chain amino acids, which in turn improves insulin resistance (56). At the same time, exercise promotes the oxidation of BCAAs, and the mechanism responsible for this phenomenon is attributed to its ability to activate branched-chain alpha-keto acid dehydrogenase (BCKDH), which catalyzes the second step of the catabolic pathway of BCAAs, and is the rate-limiting enzyme in this pathway (57). Therefore, the reduction of BCAAs induced by exercise may be an important way for exercise to modulate gut flora to improve insulin sensitivity in T2DM.

### 3.5 Exercise may improve T2DM by regulating gut flora through other pathways

Animal studies have demonstrated that long-term moderate physical activity enhances intestinal immunoglobulin A (IgA) levels and attenuates the role of B lymphocytes and CD4 + T cells in regulating the gene expression of cytokines involved in IgA production, such as IL-6, IL-4, IL-10, and TGF-*β* (26). These findings suggest that exercise may ameliorate T2DM through the enhancement of mucosal immunity and increased resistance to intestinal pathogen colonization. It has been demonstrated that exercise stimulates the muscular release of myokines, which act to enhance glucose metabolism in muscle via the activation of AMP-activated protein kinase (AMPK) and to reduce systemic inflammation (58). Furthermore, regular exercise has been shown to enhance glucose oxidation through an upregulation of glucose transporter 4 (GLUT4) transcription, leading to improved glucose uptake and a reduction in skeletal muscle insulin resistance. GLUT4 represents the primary glucose transporter subtype in skeletal muscle and is responsible for mediating glucose transport stimulated by both insulin and exercise (59). Further, exercise may increase glucose uptake, fat oxidation, and insulin secretion through a specific mechanism through the production and secretion of the inflammatory factor IL-6 by skeletal muscle tissue (60). And animal experiments have found that gut microbiota plays an important role in regulating muscle metabolism (61). These findings raise an intriguing scientific hypothesis: myokines secreted by skeletal muscles during exercise (such as IL-6) may influence local and systemic metabolism and exert a reciprocal effect on the gut via the circulatory system. This could potentially alter microbial composition or compromise intestinal barrier function, thereby establishing a feedback loop within the “muscle-gut axis.” However, this hypothesis requires direct confirmation through future research.

The progression of T2DM has been associated with an increased relative abundance of Ruminococcus in the gut microbiota. It has been suggested that transplantation of *Akkermansia muciniphila* may reduce the abundance of *R. torques*, decrease serum endotoxin levels, and promote immunomodulation, thereby potentially delaying the progression of diabetes. These findings indicate that modulation of the gut microbiota to reduce the relative abundance of *R. torques*, compared to other species such as Ruminococcus, could be beneficial for the prevention and treatment of T2DM. Supporting this concept, a 6-month randomized intervention trial demonstrated that a combination of either high-intensity interval training or moderate-intensity continuous training with a low-carbohydrate diet increased the presence of beneficial bacteria (e.g., Blautia spp.), reduced potentially harmful bacteria (e.g., Alistipes spp.), and improved overall gut microbiota composition; these changes were associated with reduced blood glucose levels (62). Collectively, these findings suggest that exercise interventions may contribute to the prevention and management of T2DM by modulating gut microbiota composition, including a relative reduction in the abundance of *R. torques*.

## 4 Discussion

In conclusion, the narrative review above found that exercise intervention can modulate the gut microbiota to alter systemic insulin sensitivity through a variety of pathways, including bile acid metabolism, short-chain fatty acid metabolism, lipopolysaccharide metabolism, and branched-chain amino acid metabolism, and subsequently contribute to the improvement of symptoms of T2DM.

However, it is worth noting that although this study analyzed the mechanism of exercise intervention on gut flora in the treatment of T2DM, the effects of different exercise modes, intensities and durations on the gut microbiota varied. Lambert et al. demonstrated via animal experimentation that compared to sedentary controls, both normal mice (*n* = 20) and T2DM mice (*n* = 20) subjected to 6 weeks of low-intensity treadmill exercise exhibited significantly increased abundance of Lactobacillus and Clostridium, while showing significantly reduced abundance of Faecalibacterium and Prevotella. Furthermore, post-intervention analysis revealed that T2DM mice retained significantly lower abundance of Bifidobacterium than normal mice (63). Bacteria such as Bacteroides spp., *Streptococcus pyogenes*, *Escherichia coli* and *Haemophilus influenzae* promote the development of diabetes, and bacteria such as *Clostridium perfringens*, *Bifidobacterium bifidum* and Coccidioides tumefaciens are able to fight against diabetes; therefore, 6 weeks of low-intensity treadmill exercise had a positive impact on T2DM mice, but the specific therapeutic effects need to be further explored.

It was observed by Charlesson B et al. that during periods of high training load, compared to periods of low load, elite rowers exhibited a decrease in gut microbiota *α*-diversity, an increase in Bacteroidetes abundance, a significantly reduced Firmicutes-to-Bacteroidetes ratio, and markedly reduced short-chain fatty acid concentrations (particularly propionate and butyrate) (64). Torquati et al. examined the impact of exercise intensity on the gut microbiota of sedentary individuals with T2DM. Their results demonstrated that 8 weeks intervention led to a significant increase in specific health-promoting bacterial communities and butyrate-producing bacteria, which was accompanied by distinct changes in gut microbial metabolic pathways. Moderate-intensity aerobic exercise combined with resistance training was shown to increase the relative abundance of Bifidobacterium, *Escherichia coli*, Bacteroides, and butyrate-producing bacteria from the order Lactobacillales and Clostridia cluster IV. Similarly, high-intensity exercise increased the abundance of butyrate-producing bacteria; however, this involved different orders (Helicobacterales, Oscillospirales) and included some less-studied genera (e.g., Methanosarcina, Neisseria). Although both regimens conferred metabolic benefits, moderate-intensity aerobic exercise combined with resistance training demonstrated superior efficacy in modulating gut microbiota structure and function (15). In a review, Borror et al. concluded that postprandial exercise is an effective strategy for improving glycemic control in individuals with T2DM. Specifically, postprandial aerobic exercise was reported to reduce the area under the glycemic curve by 3.4, 26.6% and to decrease the prevalence of 24 h hyperglycemia by 11.9, 65%. Resistance exercise was similarly found to reduce the area under the glycemic curve by 30% and the prevalence of 24 h hyperglycemia by 35% (65). Hamasaki et al. investigated an intermittent exercise intervention in individuals with T2DM and found that, compared to continuous exercise, the intermittent regimen was not only feasible and effective for glycemic control but was also superior in improving body composition, insulin sensitivity, aerobic capacity, and oxidative stress markers (66).

In the gut, nine sedentary individuals with prediabetes and seventeen sedentary individuals with T2DM underwent 3 weeks of either moderate-intensity continuous training (MICT) or sprint interval training (SIT). Both interventions were found to reduce systemic and intestinal inflammatory markers, such as tumor necrosis factor-alpha and lipopolysaccharide-binding protein. They also increased the relative abundance of *Bacteroides ovatus*, *Bacteroides thetaiotaomicron*, and the Bacteroidetes phylum ratio, while decreasing Clostridium spp. (14). Furthermore, MICT specifically reduced jejunal fatty acid-binding protein (FAU). Colonic glucose utilization (GU) was positively correlated with the Bacteroidetes phylum and negatively correlated with the Firmicutes phylum and the Firmicutes/Bacteroidetes ratio. A central challenge and opportunity in this field arises from the heterogeneity of exercise interventions. Our synthesis reveals that the impact of exercise on the gut microbiota and subsequent T2DM outcomes is not uniform but is significantly modulated by exercise prescription parameters. The evidence suggests that moderate-intensity continuous training (MICT), particularly when combined with resistance training, may be most effective in enriching classically recognized beneficial genera, such as Bifidobacterium and Lactobacillus (15, 67). This implies that distinct exercise stimuli may activate parallel or complementary microbial pathways to confer metabolic benefits. Furthermore, the concept of exercise dosage is critical, as demonstrated by findings indicating that periods of excessive training load can paradoxically reduce microbial diversity and SCFA production, even in athletes (64). This underscores a non-linear, hormetic relationship between exercise intensity/duration and gut health. Consequently, the objective is not to identify a single “best” exercise regimen but to elucidate this complex dose–response relationship.

Future large-scale, well-controlled randomized trials are urgently needed to develop personalized exercise prescriptions tailored to an individual’s baseline microbiota, metabolic phenotype, age, and gender to maximize therapeutic efficacy. Of course, prolonged excessive exercise can adversely affect intestinal function. During intense exercise, blood is redirected from visceral circulation to active muscle groups. Prolonged reduced blood flow to the intestines disrupts mucosal homeostasis, leading to damage of intestinal epithelial cells (15). Intestinal ischemia may trigger discomfort, particularly in dehydrated states, often manifesting as abdominal cramps and diarrhea, occasionally accompanied by bloody stools. Conversely, regular moderate exercise helps mitigate stress-induced intestinal barrier dysfunction (68). In a mouse model of colitis, forced treadmill exercise exacerbated inflammatory markers and outcomes, whereas voluntary wheel exercise demonstrated protective effects (69).

Specifically, *Bifidobacterium adolescentis*, Bifidobacterium christensenii, and Helicobacter macacae (probiotics or potential probiotics associated with enhanced glycolipid metabolism, insulin sensitivity, and intestinal anti-inflammatory effects) were significantly enriched in the gut microbiota of exercise-responders and formed stable symbiotic relationships. Exercise perpetuated and enhanced the beneficial intestinal microecological profile in responders, primarily through enhanced metabolic activity and stabilized symbiotic networks among beneficial microbiota. However, exercise failed to improve the gut microecology of non-responders and was even associated with a reduction in their microbial diversity. Multiple studies have demonstrated that exercise-induced modulation of gut microbiota exerts beneficial effects on T2DM. Significant gaps remain in the academic exploration of diverse exercise modalities for gut microbiota intervention and in the development of individualized research protocols accounting for factors such as age, gender, and geographic location.

## 5 Summary

In conclusion, the gut microbiota, a crucial component of the human microecosystem, is closely associated with the onset and progression of T2DM. Accumulating evidence indicates that exercise interventions can ameliorate metabolic dysfunction in T2DM patients by modulating the composition and function of the gut microbiota, offering novel insights for disease prevention and treatment strategies. However, the precise mechanisms underlying exercise-induced alterations in the gut microbiota and their contribution to T2DM improvement remain incompletely understood and are subject to ongoing debate. It is important to recognize that confounding factors, including age, gender, comorbidities, genetic background, dietary patterns, as well as exercise modality, intensity, and duration, are likely to significantly influence both the gut microbiota response to exercise intervention and its subsequent metabolic outcomes. Future research directions should prioritize the following: (1) Expanding sample sizes to enhance study reliability and generalizability. Enrollment of T2DM patients from diverse geographic regions, representing varied ages, genders, and disease durations, would facilitate a more comprehensive assessment of exercise intervention effects on gut microbiota. (2) Optimizing methodologies and employing advanced techniques to improve accuracy and sensitivity. Utilization of high-throughput sequencing, metabolomics, and other sophisticated approaches would enable in-depth analysis of structural and functional shifts within the gut microbiota, thereby elucidating the mechanistic actions of exercise. (3) Identifying optimal exercise intervention protocols. Personalized exercise regimens, tailored to individual patient conditions and needs, encompassing exercise type, intensity, frequency, and duration, should be developed to maximize therapeutic efficacy. (4) Enhancing the synergistic application of exercise with complementary therapies. Integrating exercise interventions with dietary modifications, pharmacotherapy, and other modalities to form comprehensive treatment plans holds promise for improving therapeutic outcomes and quality of life in T2DM. Investigating the impact of exercise intervention on the gut microbiota in T2DM represents a highly promising area of research. Through continuous refinement of research methodologies and optimization of therapeutic protocols, more effective and personalized treatment strategies for T2DM patients can be developed. Thus, in-depth investigation into exercise-induced modulation of the gut microbiota provides a valuable theoretical and practical foundation for T2DM management.
