# Supplementary material for: Exercise intervention regulates gut microbiota to improve type 2 diabetes: a narrative review of the mechanisms
Source: Front Nutr. 2025 Dec 24;12:1698112. doi: 10.3389/fnut.2025.1698112 (PMC12777083; doi:10.3389/fnut.2025.1698112)
Supplement: Supplementary file 1 [file Supplementary_file_1.docx]

Supplementary table 1: Scale for the Assessment of Narrative Review Articles (SANRA) details

| Scale for the Assessment of Narrative Review Articles (SANRA) | |
| --- | --- |
| 1. Justification of the article’s importance for the readership | The review addresses Type 2 Diabetes Mellitus (T2DM), a major global health challenge, and explores the novel, rapidly evolving nexus between exercise intervention, gut microbiota, and metabolic health. This topic is of high significance to researchers and clinicians in endocrinology, sports medicine, and microbiology seeking non-pharmacological therapeutic strategies. |
| 2. Statement of concrete aims or formulation of questions | The primary aim of the review is clearly stated: to summarize and discuss the physiological mechanisms by which exercise intervention modulates gut microbiota to improve T2DM. |
| 3. Description of the literature search | A systematic and reproducible literature search was conducted to minimize selection bias and ensure a comprehensive coverage of the relevant evidence. Boolean search terms included: ((exercise intervention OR physical activity) AND (gut microbiota OR gut flora) AND (type 2 diabetes OR non-insulin-dependent diabetes OR adult-onset diabetes) AND (mechanism OR physiological mechanism)) were used and searched for through Web of science, PubMed and Embase. |
| 4. Referencing | The review supports its scientific statements and conclusions with appropriate and timely citations from the primary peer-reviewed literature included in the review. |
| 5. Scientific reasoning | The review does not merely list findings but critically interprets and synthesizes evidence to build a coherent narrative on the mechanistic pathways. It acknowledges complexities and limitations within the field. |
| 6. Appropriate presentation of data | Data and complex mechanistic pathways are presented in a clear, accessible, and logical manner, primarily through the use of summary tables (in essence, the narrative synthesis) and specially designed figures. |
